# Growth of nonexudative macular neovascularization in age-related macular degeneration: an indicator of biological lesion activity

**DOI:** 10.1038/s41433-022-02282-1

**Published:** 2022-11-25

**Authors:** Yusong Wang, Junran Sun, Jiali Wu, Huixun Jia, Jingyang Feng, Jieqiong Chen, Quan Yan, Peirong Huang, Fenghua Wang, Qiyu Bo, Xiaodong Sun

**Affiliations:** 1 National Clinical Research Center for Ophthalmic Diseases, Shanghai, China; 2 Department of Ophthalmology, Shanghai General Hospital (Shanghai First People’s Hospital), Shanghai Jiao Tong University School of Medicine, Shanghai, China; 3 National Clinical Research Center for Eye Diseases, Shanghai, China; 4 Shanghai Key Laboratory of Fundus Diseases, Shanghai, China; 5 Shanghai Engineering Center for Visual Science and Photomedicine, Shanghai, China

**Keywords:** Diseases, Medical research

## Abstract

**Purpose:**

To investigate the growth of nonexudative macular neovascularization (MNV) in age-related macular degeneration (AMD) using swept-source optical coherence tomography angiography (SS-OCTA).

**Methods:**

Patients with treatment-naïve nonexudative AMD in one eye and exudative AMD in the fellow eye who underwent SS-OCTA imaging for at least 12 months were retrospectively reviewed. The MNV area measurement was quantified in eyes with treatment-naïve nonexudative MNV using ImageJ for analysing the correlation between MNV growth and the onset of exudation, as well as evaluating the consistency of the MNV growth rate during the subclinical and exudative stages. Kaplan-Meier survival analysis and logistic regression analyses were used.

**Results:**

In total, 45 eyes with treatment-naïve nonexudative AMD from 45 patients were enrolled. Treatment-naïve nonexudative MNV was identified in 21 eyes (46.67%) at baseline. The development of exudative findings was noted in eight eyes (17.78%), including six eyes with previously noted nonexudative MNV. Eyes with growing MNV (increase in area ≥50% within 12 months) had an increased risk of exudation and developed exudation earlier than eyes with stable MNV (13.60 [6.43–20.77] months versus 31.11 [26.61–35.62] months, *P* < 0.0001, Log-rank test). Consistent growth pattern of MNV lesions was further identified in eyes with growing MNV during anti-VEGF treatment.

**Conclusion:**

SS**-**OCTA allows to qualitatively and quantitatively evaluate nonexudative MNV in AMD patients. Growing MNV involved higher probabilities and a faster onset of exudation compared to stable MNV. Identifying the growth of MNV on OCTA might be helpful for establishing treatment strategies and follow-up planning.

## Introduction

Neovascular age-related macular degeneration (NVAMD) is the sight-threatening late form of AMD leading to extensive structural damage and irreversible functional loss, which is characterized by the onset of macular neovascularization (MNV) [1–3]. With the advent of swept-source optical coherence tomography angiography (SS-OCTA) technology, treatment-naïve nonexudative MNV can be detected as an asymptomatic neovascular lesion before developing exudation or leakage. The presence of nonexudative MNV in treatment-naïve eyes is commonly noted as a harbinger of exudation [4–6]. However, the duration from the nonexudative stage to the exudative stage in patients with treatment-naïve nonexudative MNV varies among patients [4–7]. In addition, patients with exudative MNV also show different responses to anti-vascular endothelial growth factor (VEGF) therapy, suggesting the biological activity of MNV is highly variable in the general population [8, 9].

The biological activity is of significance to assess the disease progression in AMD. The nonexudative MNV lesions are biologically active, with sustained lesion growth and increased MNV area, despite the fact that they are clinically inactive until they develop exudation [9–11]. Recent studies have explored the morphological parameters associated with MNV activity, including MNV area, lesion enlargement, choroid vascularity index, vessel area density, retinal pigment epithelial detachment (PED) volume, etc [6, 12, 13]. However, the clinical implications of these features in the whole course remain controversial.

The growth of MNV lesions has been recognized as an important pathological process in the subclinical and exudative periods of AMD patients, whereas the growth rate and prognosis have been reported to vary among patients [10, 13–15]. Xu et al. [10] reported that 27% treated eyes underwent MNV doubling and 46% showed modest growth (<50% increase) at one-year follow-up during anti-VEGF therapy. Forte et al. [15] reported a mean area increase of 27% during 24 months follow-up of treatment-naïve nonexudative MNV. The varied growth rates suggested different biological activity in MNV lesions. Based on this evidence, we performed a retrospective clinical study to evaluate the clinical implications of MNV growth for assessing the disease progression of AMD using swept-source optical coherence tomography angiography (SS-OCTA) imaging.

## Materials and methods

This retrospective cohort study was conducted at the Department of Ophthalmology at Shanghai General Hospital, Shanghai, China. Patients with exudative AMD in one eye and treatment-naïve nonexudative AMD in the fellow eye who were consecutively followed for at least one year using SS-OCTA from January 2017 to December 2021 were enrolled. This study was approved by the medical ethics committee of Shanghai General Hospital, and all investigations followed the tenets of the Declaration of Helsinki. Written consent was waived by the institutional review board because of the retrospective nature of the study. All data analysed were anonymized and de-identified.

### Patient selection

All patients enrolled in this study had a diagnosis of exudative AMD in one eye and treatment-naïve nonexudative AMD in the fellow eye, which was confirmed by two experienced ophthalmologists (X.S and F.W), as described below. A minimum of one-year follow-up duration using clinical examination and SS-OCTA imaging (PLEX® Elite 9000, Carl Zeiss Meditec, Dublin, CA) at each visit was required for all enrolled patients. The SS-OCTA images were obtained using the 6 × 6 mm scan pattern. Only the treatment-naïve nonexudative AMD eye of each patient was included.

The diagnostic criteria for exudative AMD were defined as evidence of MNV associated with subretinal/internal retinal fluid (SRF/IRF), serous or hemorrhagic pigment epithelial detachment (PED), or subretinal hemorrhage [16–18]. The presence of MNV was diagnosed after the review of the branching pattern of neovascular structure on the RPE-RPE fit en face angio-SS-OCTA corresponding to the vascularized PED on SS-OCT B-scans by at least 2 retina specialists (XS and FW) [18]. In the contrary, eyes with nonexudative AMD were diagnosed by the presence of pathological findings of AMD, including drusen, RPE abnormalities and/or treatment-naïve MNV lesions in absence of intra-/subretinal exudation. Eyes with nonexudative AMD were classified as either intermediate AMD (iAMD) or late AMD as previously described [6, 17, 19]. Briefly, iAMD was diagnosed when there was evidence of drusen or pigmentary abnormalities in the macula without geographic atrophy (GA) or exudation, while late nonexudative AMD was identified via the presence of GA in the absence of exudation [6, 17, 20].

The identification of treatment-naïve nonexudative MNV was based on the presence of MNV without exudative signs in the consecutive SS-OCTA B-scan images, and confirmed by at least two retina specialists (XS and FW) [19, 21–24]. To identify the neovascular lesions of the study eyes, the retina specialists carefully inspected the flow signal upon the SS-OCTA exams after the removal of the retinal vessel projections and validated the results by examining representative B-scans to localize the neovascular lesion to the appropriate layer.

The exclusion criteria included (1) a history of intraocular surgery other than cataract surgery; (2) severe media opacity; (3) excess motion artifacts; (4) MNV secondary to high myopia (≥–6.00 diopters); and (5) a history of other ocular diseases, such as advanced glaucoma, uveitis, endophthalmitis, diabetic retinopathy, and retinal detachment.

### Data collection

To analyze the characteristics of the MNV lesions, the following data were collected from the patients’ medical records, reviewed, and compared: age; gender; medical history; and clinical examinations, including best corrected visual acuity (BCVA), color fundus photography (Visucam 200 digital fundus camera, Carl Zeiss Meditec, Dublin, CA; Optos® PLC; Dunfermline, Scotland, UK), spectral-domain optical coherence tomography (SD-OCT) (Spectralis, Heidelberg Engineering, Germany), and SS-OCTA scans.

The MNV lesions were assessed based on SS-OCTA scans taken using the PLEX Elite 9000 device (Carl Zeiss Meditec Inc., Dublin, CA, USA) at baseline and at each visit. All images were carefully visualized to ascertain the correctness of segmentation and avoid the erroneous recognition of the boundaries of the retinal pigment epithelium (RPE) and RPE-fit. Manual correction was performed using the segmentation and propagation editing tool of the PLEX Elite 9000 software. For each eye, the best-quality 6 × 6 mm SS-OCTA volume scans were selected for analysis in the study.

Area measurement for MNV lesions was performed via the RPE-RPE fit segmentation of SS-OCTA images using ImageJ (NIH Image, Bethesda, MD) software. This was done by two ophthalmologists independently (YW and QB). The segmentation lines were manually adjusted when necessary to optimally visualize the lesions, and the segmentation remained consistent at each follow-up. Artifacts in the OCTA images of the outer retina were removed by the automated projection artifact removal software integrated into the instrument. An area difference greater than 30% between graders was resolved via open discussion between the graders. If no consensus could be reached, the discrepancy was adjudicated by one of the retina specialists (XS or FW).

### Grouping and lesion area measurement

The morphologic parameters of eyes with MNV lesions were reviewed at each follow-up visit. To grade the growth activity of each MNV lesion, we determined the lesion area change in the subclinical stage according to the following standards: for treatment-naïve nonexudative MNV lesions without conversion to exudation in the first year, the area change was calculated by comparing the lesion area at baseline and at the first visit after the one-year follow-up; for MNV lesions that developed exudation in the first year, MNV area change was calculated by comparing the lesion area at baseline and the visit in which exudative findings were detected. Based on the lesion area change in the subclinical stage, we subdivided the study eyes with treatment-naïve nonexudative MNV into a stable MNV group (less than 50% area growth or shrinkage) and a growing MNV group (more than 50% area growth) [10].

To further analyse the MNV activity after the onset of exudation, we calculated the maximum lesion area potential in the exudative period depending on the accessibility of the SS-OCTA scan data. The maximum lesion area potential in the exudative stage was calculated by comparing the minimum lesion area detected during anti-VEGF therapy and the maximum lesion area after that, with the follow-up durations being carefully recorded. In this case, we quantified the maximum growth capacity of the MNV lesions despite anti-VEGF therapy in order to compare the growth ability of MNV lesions in the subclinical and exudative stages.

In this study, the biological activity of MNV lesions, an intrinsic attribute of the neovascular lesion, refers to the stability of the lesion area and the tendency to develop exudation. The onset of exudation in treatment-naïve nonexudative MNV lesions was defined as the presence of sub/intraretinal fluid or a distinct serous component of the PED in any B-scan of the volume scan of the SS-OCTA image (the entire stack was reviewed), with/without clinical records of subretinal hemorrhage upon fundus examination [25, 26]. The evolution of exudative findings based on SS-OCTA images was reviewed by two experienced attending physicians independently (QB and JS). Any disagreement was resolved by open arbitration between two retina specialists (XS and FW).

### Treatment strategies

After the onset of exudation in patients with treatment-naïve AMD eyes, three initial loading doses of intravitreal anti-VEGF therapy were given followed by monthly injections until a dry macula was achieved, which was defined by the absence of any intraretinal or subretinal fluid detected by consecutive B-scan images. After that, patients were administered with pro re nata (PRN) regimens. Additional injections were administered when macular exudative changes were recurred according to experienced ophthalmologists’ decisions.

### Statistical analysis

Statistical analyses were performed using SPSS Version 24.0 (SPSS Inc, Chicago, USA). The baseline characteristics were presented as means ± standard deviations (SDs) or medians with ranges. The survival curve of the progression from treatment-naïve nonexudative MNV to exudative MNV was calculated using Kaplan-Meier survival analysis in GraphPad Prism 7. A Cox regression model was used to estimate the hazard ratio (HR) and 95% confidence intervals (95% CIs) for factors influencing the progression to exudative AMD over time. Results with *p* values of less than 0.05 were considered statistically significant.

## Results

Fifty-three eyes with complete data initially met the inclusion criteria, but five eyes were excluded due to poor OCTA image quality, and three eyes were excluded due to inadequate follow-up. A total 45 eyes from 45 patients (66.67% male) were enrolled in this study, with a mean age of 70.02 ± 6.68 years. All the included eyes were treatment-naïve, diagnosed as non-exudative AMD at baseline, and followed for at least one year using clinical examination and 6 × 6 mm scan patterns of SS-OCTA. The median follow-up duration was 24.36 ± 9.23 months (range: 12–46). Among the 45 eyes with nonexudative AMD, 21 eyes were diagnosed with treatment-naïve nonexudative MNV, and 24 were found with iAMD (drusen and/or pigmentary abnormalities).

**Fig. 1:**
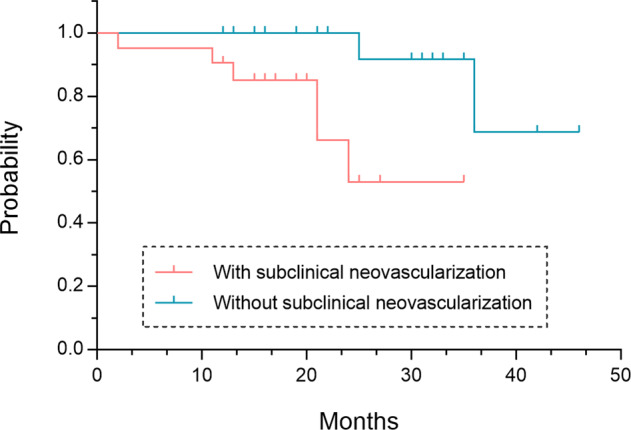
Time to develop exudative changes in NVAMD. Probability of developing exudation associated with treatment-naïve nonexudative MNV lesions among the study eyes with nonexudative AMD.

During the follow-up period, the development of exudative changes was noted in eight eyes: six of 21 eyes (28.57%) with treatment-naïve nonexudative MNV at baseline and two of 24 (8.33%) with iAMD. Eyes with treatment-naïve nonexudative MNV showed a relatively shorter subclinical stage period before conversion to the exudative stage than eyes without nonexudative MNV (Fig. 1; 27.01 [21.96–32.05] months versus 41.96 [37.11–46.81] months, *P* = .0059, Log-rank test). The hazard ratio (HR) for eyes with treatment-naïve nonexudative MNV relative to those without was 6.33 (95%CI, 1.436–27.93, Log-rank test).

Lesion area measurement was conducted in the 6 × 6 mm SS OCTA scans at each follow-up visit, with the lesion area change was calculated and analysed. The mean treatment-naïve nonexudative MNV lesion area was 1.11 ± 1.16 mm^2^ at baseline, increasing into 1.50 ± 1.66 mm^2^ at the first visit after one-year follow-up or the visit that confirmed exudation within a one-year duration (*P* = 0.008, Paired samples *t* test).

**Fig. 2:**
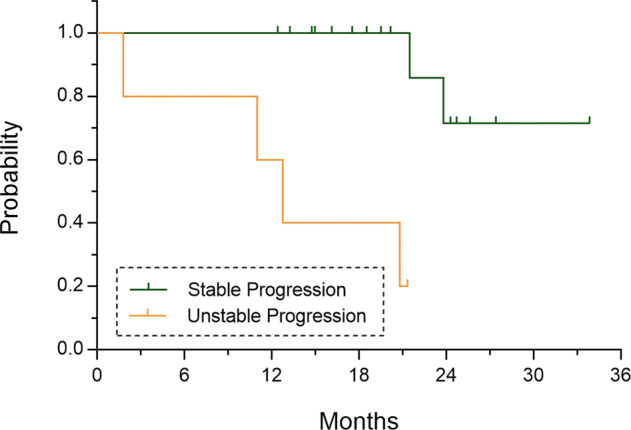
Time to develop exudative changes in eyes with treatment-naïve nonexudative MNV. Lesion area measurement was quantified and compared between baseline and the visits that first showed exudation (for the two eyes that developed exudation within one year) or the first visit after 1-year follow-up visits (for the 19 eyes without development of exudation within one year). During follow-up visits, the probability of exudation was significantly higher in eyes with growing MNV than eyes with stable MNV (*P* < 0.0001, Log-rank test).

**Fig. 3:**
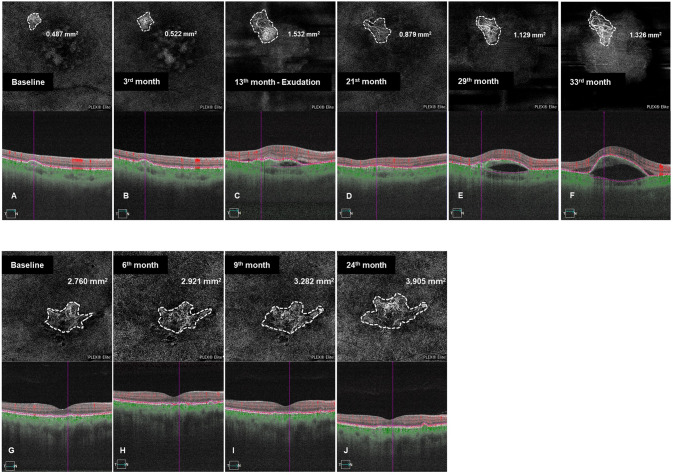
SS-OCTA 6 × 6 mm scans of the case of growing MNV lesion for 33-month follow-up and the case of stable MNV for 24-month follow-up. **A**–**F** 33-month Follow-up of growing MNV lesion. **A** SS-OCTA photographs show a treatment-naïve nonexudative MNV lesion in the patient’s right eye at baseline. **B** SS-OCTA shows that the area of the MNV lesion increased to 0.522 mm^2^ in the third month and **C** enlarged to 1.532 mm^2^, with exudation, in the thirteenth month. **D** SS-OCTA photographs of the MNV lesion detected after anti-VEGF therapy and **E** the symptomatic recurrence of exudation in the 29^th^ month. **F** SS-OCTA shows that the area of the MNV lesion increases to 1.326 mm^2^. **G**–**J** 24-month follow-up of stable MNV lesion. **G** SS-OCTA photographs show a 2.760 mm^2^ subclinical MNV lesion in the patient’s right eye at baseline. **H** SS-OCTA shows that the area of the subclinical MNV lesion increased to 2.921 mm^2^ in the sixth month and **I** enlarged to 3.282 mm^2^ in lesion area in the ninth month. **J** SS-OCTA photograph shows the lesion area increased into 3.905 mm^2^ at the last visit (the 24^th^ month).

Based on the grading criteria used in this study, five of 21 eyes with treatment-naïve nonexudative MNV were subdivided into the growing MNV group and the rest were subdivided into stable MNV group in subclinical stage (Growth rate: 96.8% ± 65.8% versus 9.3% ± 17.0%, *P* = 0.040). The baseline MNV area and median time of lesion area measurement showed no significant differences between two groups (Table 1). Growing MNV was correlated with an increased risk of exudation within a shorter time period than stable MNV (Fig. 2; 13.60 [6.43–20.77] months versus 31.11 [26.61–35.62] months, *P* < .0001, Log-rank test). The hazard ratio (HR) for exudation is 12.51 (95%CI, 1.228–127.5) in the two groups. Figure 3A–C depicts the progression of growing MNV in the subclinical stage and the development of symptomatic exudation in the 13^th^ month. The ongoing growth of the neovascular lesion was still substantially detected after anti-VEGF treatment, with the recurrence of exudation (Fig. 3D–F). In the contrast, stable MNV lesion demonstrated indistinctive growth in the first year and might remain in the nonexudative stage for rather long period (Fig. 3G–J).

**Table 1:** Lesion characteristics and medical histories in patients with treatment-naïve nonexudative MNV.

			Growing MNV	Stable MNV	*P* value
			(*n* = 5)	(*n* = 16)	
Age (years)			72.14 ± 0.48	67.83 ± 1.37	0.102^
Baseline MNV Area (mm^2^)			1.59 ± 1.46	0.95 ± 1.05	0.294^
Duration of lesion measurements (months)			12.00 ± 6.81	16.03 ± 3.65	0.097^
Personal History	Alcohol	No	3	12	1
		Yes	2	5	
	Cigarettes	No	1	14	0.021*
		Yes	4	3	
Medical History	Hypertension	No	2	8	1
		Yes	3	9	
	Diabetes	No	4	14	1
		Yes	1	3	
	Cerebral Infarction	No	3	16	0.117
		Yes	2	1	

The asterisk (*) indicated statistical significance in two groups, ^Unpaired Student *t* test, otherwise Fisher’s exact test was used.

*MNV* macular neovascularization.

**Fig. 4:**
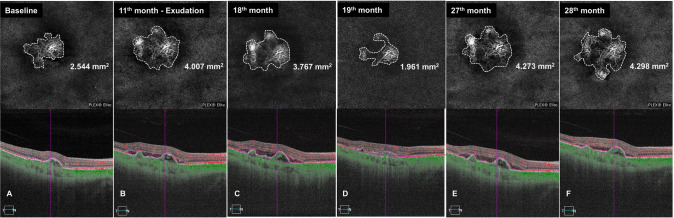
SS-OCTA 6 × 6 mm scans of a growing MNV that developed exudation after 11 months with 28-month follow-up visits. **A** SS OCTA photograph shows a 2.544 mm^2^ treatment-naïve nonexudative MNV lesion in the patient’s left eye at baseline. **B** SS OCTA shows that the area of subclinical MNV lesion increases to 4.007 mm^2^ in eleven months and develops exudation. **C** SS OCTA shows the area of nonexudative MNV lesion as 3.767 mm^2^ before the first intravitreal anti-VEGF treatment and **D** the lesion shrinks to 1.961 mm^2^ two weeks after anti-VEGF treatment. **E** SS OCTA shows that the area of subclinical MNV lesion re-enlarges to 4.273 mm^2^, and **F** to 4.298 mm^2^ eight and nine months after the anti-VEGF treatment, respectively.

We further reviewed the SS-OCTA scans of MNV lesions that had been followed for at least one year after developed exudation (four eyes; three with growing MNV and one with stable MNV). The patients were administrated with three consecutive monthly anti-VEGF injections followed by the PRN (3+PRN) regimen after the detection of developing exudation. Three eyes in the growing MNV showed sustained growth of the neovascular lesion and higher growth capacity than stable MNV during anti-VEGF treatment intervals (Supplementary table 1). As shown in Fig. 3A–F and Fig. 4, the growing MNV lesion underwent sustained enlargement despite anti-VEGF treatment and showed a recurrence of exudation.

In order to investigate the specific characteristics associated with the growing progression of treatment-naïve nonexudative MNV lesions, we collected the personal and medical histories of our patients. We found that patients with a history of smoking had a higher risk of developing enlargement (*P* = 0.021, Fisher’s precision probability test), while a medical history of alcohol use, hypertension, diabetes, or cerebral infarction showed no significant correlation with the lesion growth of treatment-naïve nonexudative MNV lesion in our patients (Table 1).

## Discussion

In this study, we identified treatment-naïve nonexudative MNV in 21 (46.67%) of 45 fellow eyes from patients previously diagnosed with exudative AMD using SS-OCTA. Eyes with treatment-naïve nonexudative MNV showed a higher risk of developing exudation than eyes without (hazard ratio: 6.33). In fact, MNV growth was commonly detected in treatment-naïve nonexudative MNV lesions; however, the lesion growth rate was diverse, ranging from -0.05% to 212.73%. Furthermore, the risk of exudation was greater for eyes with growing MNV than eyes with stable MNV. Among cases of subclinical nonexudative MNV in which exudation occurred, the sustained growth of MNV lesions was detected during anti-VEGF treatment. These results suggest that the growth rate of MNV in the subclinical stage may indicate the innate activity of the neovascular lesions, which may be associated with exudation risks and may not be altered by initial anti-VEGF therapy presumably.

The nonexudative growth of neovascular lesions has been commonly identified in previous studies in NVAMD. In Priore’s study, researchers used a double reciprocal plot as a model to determine the pattern of the enlargement of MNV lesions and suggested that MNV lesions would eventually enlarge to a size of 10.6 disc areas without treatment [27]. Xu’s long-term study discovered that the progressive growth of CNV was a pathological process in the majority of lesions (80%) during anti-VEGF therapy, with a mean growth as 0.20 ± 0.38mm^2^ per year (*P* = 0.001) [10]. These studies suggested that, although anti-VEGF therapy may limit the absolute growth rate of neovascular lesions, a sustained pattern of growth involving repeated cycles of vascular pruning and reproliferation may be the innate rule [28]. However, the underlying pathological mechanism of nonexudative growth remains unclear.

Several hypotheses were discussed regarding the potential characteristics associating with MNV growth activity in certain patients. Teo et al. [29] demonstrated that patients with systemic characteristics such as hyperlipidaemia or elevated triglycerides had more risk of progression to exudation and baseline MNV size was also a morphologic factor in eyes with nonexudative MNV. Ueta et al. [30] reported that PCV lesions may have more of a tendency to develop exudation than typical AMD in Japanese patients. Additionally, based on Sacconi’s study, MNVs located around the atrophic area of RPE cells are more susceptible to growth in lesion size, which may be due to the greater ischemic damage to the choriocapillaris around the GA area and the increased production of VEGF by the surviving RPE cell [14, 31].

It is generally recognized that the growth of nonexudative neovascular lesions may be the body’s attempt to recapitulate the choriocapillaris closer to the RPE and support RPE function with vital nutrition [30]. The gradually progressive impairment of the integrity of the RPE and the choroidal vascular architecture left the subclinical lesions with time windows for gradual enlargement before exudation. Therefore, as inducers of angiogenesis in retinal microenvironment, patients with systemic diseases, such as microangiopathy or hypoxia, may be more prone to neovascular enlargement [32, 33]. Interestingly, according to the medical records of our patients, smoking history showed a statistical correlation with lesion growth in MNV lesions (*P* = 0.021, Fisher’s precision probability test). Further investigations are needed to explore these characteristics as risk factors for lesion growth.

Currently, the clinical recommendation for eyes with treatment-naïve nonexudative MNV is frequent follow ups [5, 6]. Several case reports have suggested that patients with treatment-naïve nonexudative MNV lesions are among those AMD patients that may remain stable and non-symptomatic for a long period and that such a situation could even be beneficial in terms of protecting against atrophy [4, 14, 34]. Aggressive treatments for such lesions are undoubtedly excessive. For eyes with exudative MNV, therapeutic strategies (e.g., PRN therapy, treat and extend (T&E) therapy, or a fixed regimen) have been introduced to reduce the treatment burden, but there is still a need for evidence regarding the optimal treatment strategies for NVAMD patients. In this study, MNV growth during the nonexudative period might suggest the biological activity of MNV lesions and might give a hint of whether the lesion should be monitored more frequently in the subclinical and exudative stage, however, further prospective studies are still needed to confirm this hypothesis.

Also, MNV monitoring is of significance in guiding treatment during the exudative period. A number of studies have reported the nonexudative growth of CNV lesions in AMD eyes during anti-VEGF treatment [35–38]. Kwon et al. [13] discovered that CNV growth without fluid was more likely to precede exudative recurrence and the worsening of response to anti-VEGF treatment. However, Shen et al. [12] reported that MNV growth was not correlated with near-term exudation. This evidence shows that long-term follow up is critical in evaluating the clinical implications of MNV growth.

Our study is limited by a relatively small cohort size, a deficiency of long-term follow ups, and the retrospective design. In this study, we included a relatively small number of patients from a single institution and lacked precise follow-up schedule for all patients due to the uncertain need for anti-VEGF therapy in their exudative AMD eyes. Prospective studies are needed to further evaluate the clinical value of nonexudative MNV growth in determining treatment strategy.

In conclusion, SS-OCTA provides a non-invasive, simple, and quick detection method for monitoring MNV growth during the subclinical period, as well as the exudative period. Patients with growing MNV suffer from a higher probability of and a shorter time period before the onset of exudation. The growth activity during the exudative period was consistent with that during the subclinical period, suggesting that growth activity represents the nature of an MNV lesion and may not be altered by initial anti-VEGF therapy presumably. Finally, MNV growth activity in the nonexudative stage may give a hint to the follow-up plans of AMD patients, but further prospective studies are required to confirm this hypothesis.

## Summary

### What was known before

- Since the advent of swept-source optical coherence tomography angiography (SS-OCTA), treatment-naïve nonexudative macular neovascularization (MNV) has been detected before the onset of exudation in eyes with age-related macular degeneration (AMD). The presence of treatment-naïve nonexudative MNV may predispose to the development of exudative changes. However, the duration from nonexudative state to the onset of exudation in eyes with MNV is varied.

### What this study adds

- Based on the lesion area change in subclinical stage, we subdivided the study eyes with subclinical MNV into stable MNV group (less than 50% area growth or shrinkage) or unstable MNV group (more than 50% area growth) to describe the biological activity of neovascular lesions. Eyes with unstable MNV correlated with more risks of exudation and developed exudation earlier than eyes with stable MNV. Sustained growth of MNV was identified in unstable MNV lesions after developed exudation despite ongoing anti-VEGF therapy. As an indicator of the MNV lesion activity, lesion growth might contribute to establishing treatment strategies and follow-up planning for AMD patients.

## Supplementary information

Supplementary Table 1

## Data Availability

All data generated or analysed during this study are available from the corresponding author upon reasonable request.
